# A Comparison of Four-Year Health Outcomes following Combat Amputation and Limb Salvage

**DOI:** 10.1371/journal.pone.0170569

**Published:** 2017-01-25

**Authors:** Ted Melcer, Jay Walker, Vibha Bhatnagar, Erin Richard, V. Franklin Sechriest, Michael Galarneau

**Affiliations:** 1 Department of Medical Modeling, Simulation, and Mission Support, Naval Health Research Center, San Diego, California, United States of America; 2 VA San Diego Healthcare System, San Diego, California, United States of America; 3 Department for Family Medicine and Public Health, University of California San Diego, San Diego, California, United States of America; 4 VA Minneapolis Healthcare System, Minneapolis, Minnesota, United States of America; Northwestern University, UNITED STATES

## Abstract

Little research has described the long-term health outcomes of patients who had combat-related amputations or leg-threatening injuries. We conducted retrospective analysis of Department of Defense and Department of Veterans Affairs health data for lower extremity combat-injured patients with (1) unilateral amputation within 90 days postinjury (early amputation, *n* = 440), (2) unilateral amputation more than 90 days postinjury (late amputation, *n* = 78), or (3) leg-threatening injuries without amputation (limb salvage, *n* = 107). Patient medical records were analyzed for four years postinjury. After adjusting for group differences, early amputation was generally associated with a lower or similar prevalence for adverse physical and psychological diagnoses (e.g., pain, osteoarthritis, posttraumatic stress disorder) versus late amputation and/or limb salvage. By contrast, early amputation was associated with an increased likelihood of osteoporosis during the first year postinjury. The prevalence of posttraumatic stress disorder increased for all patient groups over four years postinjury, particularly in the second year. The different clinical outcomes among combat extremity injured patients treated with early amputation, late amputation, or limb salvage highlight their different healthcare requirements. These findings can inform and optimize the specific treatment pathways that address the physical and psychological healthcare needs of such patients over time.

## Introduction

Previous studies indicated that extremity injuries accounted for approximately half of combat wounds sustained by US service members in Iraq and Afghanistan conflicts [1,2]. For patients with the most severe leg injuries, treatment required either amputation or leg-threatening injuries without amputation (hereafter referred to as limb salvage) through reconstructive surgeries [3]. Patients with combat-related amputation and limb salvage continue to present new treatment and rehabilitation challenges to healthcare providers at both military and Department of Veterans Affairs (VA) facilities [4–8].

There has been little longitudinal research comparing health outcomes following amputation or limb salvage [5,6]. Previous research has demonstrated differences between patients treated with unilateral lower extremity amputation and those with limb salvage with respect to the likelihood of wound complications and adverse psychological outcomes in the first two years after injury [5]. However, it is unclear whether these differences persist beyond the first two years. In the long-term, severe lower extremity injuries alter normal gait and may limit mobility. As a result, secondary health complications (i.e., pain, osteoarthritis, hypertension, and posttraumatic stress disorder [PTSD]) may arise and exacerbate the physical disability caused by the initial limb injury [9]. Therefore, in order to inform development of clinical pathways that improve treatment and rehabilitation of these patients, there is a need to quantify and compare long-term health outcomes following amputation and limb salvage.

A recent study followed the clinical diagnoses for nearly 700 patients following combat amputation or limb salvage that were recorded at military treatment facilities over their first two years post-injury, which includes over 95% of all US service members who sustained combat related lower limb amputations between 2001 and 2008 [5]. Early amputation (within 90 days of injury) was generally associated with a reduced likelihood of diagnoses for infection, PTSD, substance abuse, and mood disorders relative to limb salvage or late amputation [5]. In contrast, early amputation was associated with an increased likelihood of developing anemia and heterotopic ossification relative to limb salvage or late amputation. However, most patients did not have military health data for follow-up after two years, usually because they had transitioned to the VA healthcare system. In general, there has been little research combining existing DOD and VA data to describe patient health outcomes over an extended period of time following combat injury [6].

The first objective of this study was to follow clinical diagnoses for 625 individuals with lower extremity amputation or limb salvage [5] for four years postinjury by integrating military and VA health data. We expected that a large majority of these individuals were treated at military and/or VA facilities for many years after injury [10]. The second objective was to quantify and compare patient prevalence of wound complications, other physical health complications, and adverse psychological diagnoses. Based on previous findings [5,6], we hypothesized that early amputation would be associated with reduced prevalence of adverse health diagnoses compared with late amputation and/or limb salvage [5].

## Methods

### Data Sources

Injury-specific data were obtained from the Expeditionary Medical Encounter Database (EMED) [11]. This database includes a clinical encounter form designed for research purposes to capture casualty records primarily at forward treatment facilities in Iraq and Afghanistan. The EMED forms contain notes by treating providers, including surgical and evacuation reports at Role 2–5 care facilities such as Landstuhl Regional Medical Center and U.S. military treatment facilities (e.g., Walter Reed/Bethesda, San Antonio, and San Diego). These records provide detailed descriptions of serious extremity injuries (e.g., fractures, soft tissue, and vascular injuries) and associated treatments through the continuum of military casualty care. Abbreviated Injury Scale (AIS) scores were assigned by EMED clin icians [11] and used to calculate Injury Severity Scores (ISSs) [12]. For patients with amputations not captured by the EMED, ISSs were obtained from the Joint Theater Trauma Registry [13]. Both projects employ experienced combat trauma nurses with extensive AIS coding experience, which is associated with reliable scoring [14].

#### Military health data

Patients and their associated anatomical levels of amputations and subsequent adverse health outcomes were identified by searching military health databases [15] for *International Classification of Diseases*, 9th Revision, Clinical Modification (ICD-9-CM) diagnostic codes. These databases include inpatient and outpatient service record files via TRICARE Management Activity files. Records were merged with those from the Armed Forces Health Longitudinal Technology Application, which is routinely generated for inpatient and outpatient encounters by credentialed providers at military treatment facilities and government-reimbursed private clinics. The patient identifiers were then encrypted, password protected, and sent electronically to VA investigators using public key infrastructure (PKI). This allowed patients to be identified and tracked in VA health databases.

#### Department of veterans affairs health data

All patients in the present study first were identified in the military health databases as described. VA data, including outpatient diagnoses (ICD-9-CM codes), were extracted from the Corporate Data Warehouse, a national repository of data from the Veterans Health Administration (VHA) facility electronic medical record system, as well as several other VHA clinical and administrative systems [16]. Data extracts were prepared by a VA Informatics and Computing Infrastructure data manager and downloaded to a secure local VA San Diego Healthcare System server using a SAS software interface (SAS Institute, Inc., Cary, NC) [17]. Data were then merged with de-identified and unique subject numbers supplied by Naval Health Research Center (NHRC) investigators and stripped of all Health Insurance Portability and Accountability Act identifiers. As previously mentioned, the de-identified data were then encrypted, password protected, and sent electronically to NHRC investigators using PKI. Health data obtained from both military and VA treatment facilities were captured from October 2001 to December 2011.

Final integration and analysis of military and VA physical and psychological health outcomes identified by ICD-9-CM codes (e.g., infections, PTSD, osteoarthritis) were completed by NHRC investigators. However, military and VA healthcare use (e.g., frequency of outpatient clinic visits) were analyzed separately because they were based on different database reporting systems. Military outpatient clinic use data came from the Medical Expense Performance and Reporting System and VA healthcare use data came from VA-specific stop codes.

#### Study population and patient identification

Patients who died from their wounds and those with brain or spinal injuries that caused extremity paralysis were excluded. After exclusions, we identified 954 U.S. military personnel who sustained major limb amputations (excluding fingers and toes) or leg-threatening injuries without amputation (limb salvage) during the Afghanistan or Iraq conflicts from 2001 through 2008.

The population count for patients who had combat-related amputations in Iraq and Afghanistan has been consistently reported by several research groups [18–20]. The total counts are based on reviews of casualty records and/or searches for relevant ICD-9 codes using military health databases including the EMED [11,18–20]. We searched the EMED to identify 847 patients with limb amputations. This count was within one percent of an independent military count of 851 reported elsewhere [19]. For the present study, we included patients with a unilateral lower extremity amputation (*n* = 518). Lower limb amputation was identified using ICD-9-CM codes (i.e., 896, 897). We recorded anatomic level of amputation, including above the knee (i.e., transfemoral or higher level including hip disarticulations) or below the knee (i.e., transtibial or lower level including ankle/foot or partial foot). Patients with amputations during the first 90 days postinjury were classified as early amputation. Patients who had amputations more than 90 days postinjury were classified as late amputation [5,21]. A separate group of 107 patients had unilateral leg-threatening injuries without amputation (limb salvage) as defined below. These 625 patients with either lower limb amputation or lower extremity limb salvage comprised the cohort for the present study.

Presently, there is no established algorithm for identifying the population of patients who underwent combat related limb salvage, such as searching for a particular set of ICD-9-CM codes in military health system databases. Identification of limb salvage injuries (e.g., complex fractures) is labor-intensive and requires extensive and expert clinical review of casualty records. Given the large number of patients who sustained significant extremity trauma in Iraq and Afghanistan, it was not practical to review the files of all of these individuals. Therefore, as previous studies have done [5,6,22], we identified a sample of patients who underwent limb salvage (from the EMED). We used well-established criteria based on previous civilian and combat trauma studies to identify the limb salvage injuries [5,6,22–24].

An experienced military orthopedic surgeon (primary reviewer) reviewed casualty records for a sample of 200 patients selected at random from the EMED with a serious lower extremity injury (AIS ≥ 3). We found no significant differences in ISS or age between the 200 patients reviewed (mean ISS, 13.2; median ISS, 10.0; mean age, 24.8 years; median age, 22.0 years) and the remaining 265 patients in the EMED who also had serious extremity injuries (AIS ≥ 3) but were not reviewed (mean ISS, 14.2; median ISS, 10.0; mean age, 24.2 years; median age, 22.0 years; *p*’s > 0.10, two-tailed t test of means, after log transformation to normalize distributions, or nonparametric two-tailed Mann-Whitney U-test).

The process for identifying patients with limb salvage injuries has been previously described [5] and is as follows. First, patients had to meet limb salvage injury criteria including one or more of the following: complex fractures (Gustilo-Anderson classification for open fractures [23] grades IIIC, IIIB, and selected IIIA), vascular injuries, major soft tissue injuries, and/or severe foot injuries. Major soft tissue injuries were degloving, avulsion, or severe crush injury, and were usually associated with extensive contamination. Severe foot injuries were Grade 3B, 3C, or Grade 3 intra-articular calcaneus fractures involving the distal tibia (pilon fracture). Second, the reviewing surgeon assigned a limb salvage score from 0–10 to indicate his judgment that the patient had limb salvage injuries (0 = definitely not limb salvage, 5 = possibly limb salvage, 10 = definitely limb salvage). The limb salvage group consisted of patients who met limb salvage injury criteria and who had limb salvage scores of 5 or greater. For 91% of records reviewed (181 of 200), the surgeon’s identification of the presence or absence of objective injury criteria in the casualty records agreed with his limb salvage scores. For example, scores of less than 5 typically were associated with no identifiable limb salvage injury criteria in the casualty records. By contrast, scores of five or greater typically were associated with positive identification of one or more injury criteria in the casualty records (e.g., grade IIIB fracture).

As described, the EMED provided detailed Role 2–5 casualty records, which the surgeon reviewed to identify the specific injury criteria (e.g., grade IIIC fracture) [8,11,20–22]. The reviewing surgeon noted cases in which there was insufficient detail to identify the presence or absence of limb salvage criteria (less than 5% of 200 cases reviewed). A second reviewing surgeon was available for a small sample of 13 cases. The primary and secondary reviewing surgeons agreed on limb salvage criteria (e.g., grade 3B fracture) for 12 of these 13 cases. Finally, the primary reviewing surgeon judged whether injuries were limb salvage based on either an acute threat to the extremity (e.g., dysvascular limb) or if limb amputation was a practical option.

### Research Design

This was a retrospective analysis of military and VA health records up to 48 months postinjury (of the 625 patients in the cohort, 60 had only 36 months of follow-up data because they were injured in 2008 and the most recent records were extracted through 2011). Mechanisms of injury were categorized as blast or nonblast (e.g., gunshot wound). ISS was calculated as previously described. The codes used to identify traumatic brain injury (TBI) have been described previously [25]. Results were analyzed for three types of postinjury complications: wound complications, physical health complications, and psychological complications.

#### Wound complications

Wound complications selected for study were those that required prolonged surveillance following serious leg injuries. This included a category for any infection as defined by ICD-9-CM codes (postoperative: 998.50–998.59; bacterial: 014.0–014.19, 014.83–014.85; cellulitis: 682.0–682.99; osteomyelitis: 730.0–730.99; chronic infection of amputation stump: 997.62; infection/inflammation due to device: 996.60–996.69; septicemia: 038.0–038.99). Three other types of wound complications were selected: deep vessel thrombosis with pulmonary embolism (453.40–453.99, 415.10–415.19), nonunion fractures (733.80–.82) and nonhealing wound (998.83). Some analyses looked at osteomyelitis (730.0–730.99) by itself. We did not report on heterotopic ossification because it has been well studied previously. We also noted there were some inconsistencies in diagnosis of this condition between 2001 and 2008.

#### Physical health complications

Other physical health complications may have resulted directly from the injury, such as a blast causing immediate joint or nerve damage. There also may be secondary complications such as altered gait, which may cause pain and osteoarthritis to develop over time [9]. The ICD-9-CM codes for physical health complications for the present study were identified by the EMED and clinicians treating patients with amputations [11]. These included late effects of musculoskeletal injury (905.0–905.9), osteoarthritis (715.0–715.3, 715.8, 715.9), and osteoporosis (733.0), as well as lumbago/back pain (724.1–724.9) and hypertension (401–405) (the diagnosis for late effects of musculoskeletal injury is generally used to indicate long-term effects related to an acute injury but may present at any point after injury). In addition, a general category of pain diagnoses was created which consisted of unspecified, acute, or chronic pain (338.0, 338.11, 338.18–338.21, 338.29), joint pain (719.4), cervical pain (723.1), back pain (724.1–724.9), and limb pain (729.5).

#### Psychological disorders

Psychological diagnostic codes were grouped as adjustment, anxiety, mood, PTSD, substance abuse, and other psychological diagnoses as described previously [26]. PTSD cases were defined as two or more separate healthcare encounters at which the ICD-9-CM diagnostic code of 309.81 was recorded at least 30 days postinjury. Preinjury military psychological records indicated whether each patient showed at least one preinjury diagnosis.

### Statistical Analyses

The following statistical methods were used to analyze each of the three types of postinjury complications:

#### Four-year prevalence (univariate analysis)

The overall prevalence was calculated for any occurrence of a diagnosis during the four-year postinjury period. Means and medians were used to summarize demographic variables. Chi-square or Fisher’s exact tests were used as appropriate to compare frequency data (e.g., number of patients with PTSD) for the amputation and limb salvage samples.

#### Four-year prevalence (multivariate analysis)

Logistic regression analyses were conducted over four years postinjury to determine whether any injury group (early amputation, late amputation, or limb salvage) was significantly associated with diagnostic outcomes when adjusting for covariates. Odds ratios (ORs) and confidence intervals were calculated for adjusted age (<25, 25–34, and 35+ years), log ISS, mechanism of injury (blast or nonblast), injury year (2001 through 2005 or 2006 through 2008), injury location (above knee or below knee), and preinjury psychological diagnosis. Year of injury was categorized as 2001 through 2005 or 2006 through 2008 because the period of injury was correlated with changes in operational tempo (e.g., the Iraq surge, 2007), which may have affected health outcomes [5].

#### Longitudinal results (pairwise comparisons)

The prevalence of specific diagnoses (e.g., infections or PTSD) was calculated separately for each of the first four years postinjury as well as the significant differences between the patient groups over time.

#### Longitudinal results (repeated measures)

Repeated measure analyses were conducted for four years postinjury using longitudinal random intercept models (SAS PROC GLIMMIX for generalized linear mixed models) to evaluate associations between unilateral injury group, TBI, log ISS, age (≤25 or >25), year after injury (years 1, 2, 3, and 4), and the likelihood of having a physical or psychological diagnosis occurring within the first four years post-injury. For significant interactions between injury group and year after injury, follow-up regressions were conducted and results are described in the text separately. An unstructured covariance matrix was selected based on the lowest Akaike information criterion [27].

## Results

Table 1 shows the demographic and injury characteristics for the study sample. The subjects were relatively young males with moderate to serious ISSs. The early amputation group had more severe injuries in comparison to the late amputation or limb salvage groups. Specifically, the early amputation group had significantly higher ISSs compared to the late amputation or limb salvage groups (*p*’s < 0.05) and a higher percentage of patients with blast injuries than the two comparison groups (*p*’s < 0.05). The early amputation group had a higher prevalence of above knee amputations (37%) compared to the late amputation (12%) and limb salvage (23%) patients (*p*’s < 0.05).There were no statistically significant differences in the prevalence of TBI or preinjury psychological diagnoses among the three groups.

**Table 1:** Injury Characteristics of Patients who Underwent Amputation or Limb Salvage.

Injury Characteristics	Early Amputation	Late Amputation	Limb Salvage
	(≤90 Days Postinjury)	(>90 Days Postinjury)	(No Amputation)
	Unilateral	Unilateral	Unilateral
	*n* = 440	*n* = 78	*n* = 107
**Age (median)**	24.2	24.3	22.8
**ISS (mean/median)**	16^*^/14	12^*^/10	14/10
**Mechanism of injury (% blast)**	89^*,†^	74^*^	68^†^
**Amputation or limb salvage Injury location (%)**			
** Above the knee**	37	12	23
** Below the knee**	63^*,†^	88^*,‡^	77^†,‡^
**TBI (%)**	34	27	29
**Preinjury psych diagnosis (%)**	10^†^	9	6^†^

Amputation times (days postinjury) per patient were 0: *n* = 523; 1–30: *n* = 58; 31–90: *n* = 6; 91–180: *n* = 10; 181–360: *n* = 26; 361–730: *n* = 41; and >730: *n* = 7).

Differences between unilateral groups (*p* < 0.05 chi-square or Fisher’s exact test as appropriate) were *early amputation versus late amputation, †early amputation versus limb salvage, and ‡late amputation versus limb salvage.

The primary leg-threatening injury among patients who underwent limb salvage: G-A grade IIIC (8%), G-A grade IIIB fractures (56%), G-A grade IIIA (8%), major soft-tissue injuries (14%), penetrating vascular wound (7%), and severe foot/ankle injuries (8%). Over 50% had more than one of these injury criteria.

G-A = Gustilo-Anderson classification for open fractures; ISS = Injury Severity Score, TBI = traumatic brain injury.

### Wound Complications

#### Four-year prevalence

Table 2 shows the prevalence of selected wound complications over four years postinjury for the early amputation, late amputation, and limb salvage groups. The limb salvage group had a significantly lower prevalence of infections (63%) compared to the early (75%) and late amputation (82%) groups (*p*’s < 0.05). Table 2 also shows that outside of infections, the early amputation group generally had a lower prevalence of wound complications than the late amputation group as well as significantly fewer non-union fractures (10%) than both comparison groups. Of particular note, the late amputation group had the highest likelihood of osteomyelitis (53%) and non-union fractures (44%; *p*’s < 0.05). The prevalence of DVT/PE did not differ significantly among the three groups.

**Table 2:** Percent of Patients who Underwent Amputation and Limb Salvage with Wound, Physical, and Psychological Health Complications.

Injury Characteristics	Early Amputation	Late Amputation	Limb Salvage
	*n* = 440	*n* = 78	*n* = 107
**Wound complications**			
** Infection**	75%†	82%‡	63%†,‡
** Osteomyelitis**	35%*	53%*,‡	36%‡
** Nonunion Fractures**	10%*,†	44%*	35%†
** Non-healing Wounds**	12%*	24%*	19%
** DVT/PE**	16%	17%	18%
**Physical Health Complications**			
** Any Pain-related**	92%	99%	93%
** Musculoskeletal**	98%	99%	100%
** Late Effects—Musculoskeletal Injuries**	34%*,†	65%*,‡	48%†,‡
** Osteoarthritis**	10%*,†	21%*	21%†
** Osteoporosis**	16%^†^	8%	2%†
** Hypertension**	15%	14%	9%
** Lumbago**	48%	56%‡	36%‡
**Psychological Complications**			
** Any Mental Health**	89%	89%	82%
** PTSD**	49%	58%	51%
** Anxiety**	40%*	54%*	40%
** Mood**	39%*	55%*,‡	36%‡
** Adjustment**	46%	53%	43%
** Substance Abuse**	21%*	31%*	27%

Differences between unilateral groups (*p* < 0.05 chi-square or Fisher’s exact test as appropriate) were *early amputation versus late amputation, †early amputation versus limb salvage, and ‡late amputation versus limb salvage.

PTSD = post-traumatic stress disorder, DVT/PE = deep vessel thrombosis/pulmonary embolism.

Table 3 shows the results of logistic regression models used to evaluate associations between injury groups and prevalence of a wound complication during the first four years postinjury. After adjusting for covariates (e.g., ISS, blast injury), the late amputation group was over three times more likely to have diagnoses for any infection, and over twice as likely for osteomyelitis than the limb salvage group (significant odds ratios and 95% confidence intervals shown in Table 3). Early amputation demonstrated a reduced likelihood for osteomyelitis compared to limb salvage in the overall four-year model. Diagnoses of deep vein thrombosis/pulmonary embolism (DVT/PE) and non-healing wounds were not significantly associated with any particular injury group. While there were no significant associations between blast injury and any of the wound complications studied here, increasing ISS was associated with an increased likelihood of any infection, osteomyelitis, and non-healing wound.

**Table 3:** Summary Results for Overall (4 Year) Regression Models for Wound and Other Physical Health Complications.

Independent Variables	Postinjury Outcomes Variables OR (95% CI)							
	Wound Complications				Physical Health Complications			
	*Any Infection	*Osteomyelitis	Nonhealing Wound	DVT/PE	*Any Musculoskeletal	Pain	Osteoarthritis	Osteoporosis
**Injury Group**								
** Limb salvage (ref)**	–	–	–	–	–	–	–	–
** Early amputation**	NS	0.46	NS	NS	0.40	0.59	0.32	10.41
		(0.23–0.91)			(0.23–0.70)	(0.39–0.88)	(0.16–0.64)	(2.44–44.49)
** Late amputation**	3.60	2.46	NS	NS	NS	2.11	NS	NS
	(2.09–6.15)	(1.04–5.82)				(1.19–3.74)		
**Blast (vs. no blast, ref)**	NS	NS	NS	NS	NS	1.88	2.81	NS
						(1.17–3.03)	(1.08–7.32)	
**ISS (log)**	1.34	1.43	1.50	1.43	1.23	NS	NS	NS
	(1.16–1.54)	(1.15–1.77)	(1.22–1.85)	(1.15–1.77)	(1.05–1.44)			
**Age, year**	NS	1.00	NS	1.04	1.04	NS	1.08	NS
		(1.00–1.08)		(1.00–1.08)	(1.00–1.07)		(1.03–1.13)	
**Injury year (2001/5 vs. 2006/8, ref)**	NS	0.47	NS	0.47	0.59	0.55	NS	NS
		(0.29–0.75)		(0.29–0.75)	(0.43–0.84)	(0.40–0.75)		

TBI and injury location (above vs. below knee) were not significant in the models.

* Variables with significant injury group × time interactions (see text for summary of interactions).

Nonunion fracture: Results based on very few cases for early amputation. Early amputation had significantly lower ORs than limb salvage. Late amputation ORs not significantly different from limb salvage. Increased ISS associated with significantly increased ORs.

Late effects of injury: early amputation (OR = 0.49 [95% CI, 0.28–0.85] and had significantly reduced OR relative to limb salvage. Increasing ISS also was associated with significantly increased OR (OR = 1.46 [95% CI, 1.2–1.76]) relative to limb salvage.

CI = confidence interval, DVT/PE = deep vessel thrombosis/pulmonary embolism, ISS = Injury Severity Score, NS = not significant, OR = odds ratio, TBI = traumatic brain injury.

#### Longitudinal results

**Fig 1:**
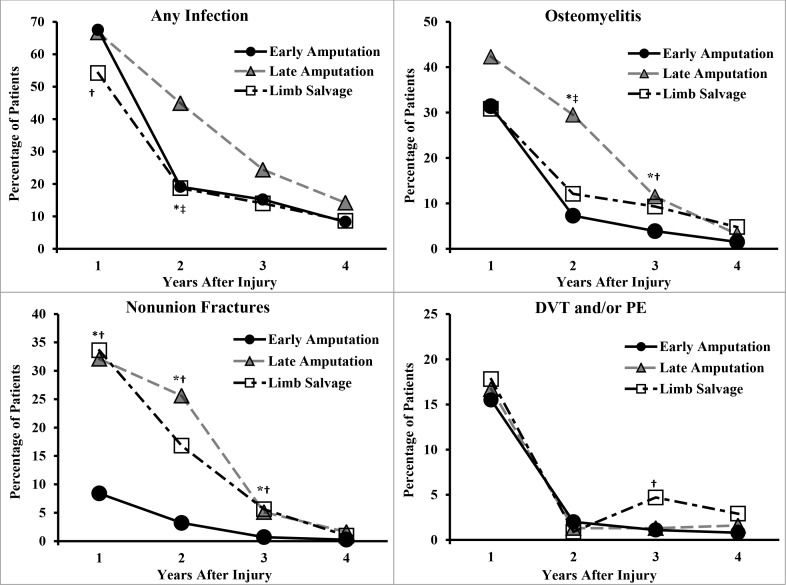
Prevalence of Selected Wound Complications by Time After Injury. Statistically significant difference between groups, using chi-square or Fisher’s exact test as appropriate, p < 0.05 were *early amputation versus late amputation, †early amputation versus limb salvage, and ‡late amputation versus limb salvage. (Table 3 shows where injury group was significantly associated with outcomes after adjusting for covariates). DVT = deep vessel thrombosis, PE = pulmonary embolism.

Fig 1 highlights several important results based on univariate analyses. First, while all groups generally had a relatively high prevalence for selected wound complications in the year after injury, the overall prevalence declined significantly over the four years (*p*’s < 0.01). Second, the prevalence of infections and nonunion fractures remained relatively high through the second year. Third, late amputation was associated with a significantly higher prevalence for both osteomyelitis and any infection compared to the limb salvage and early amputation groups, primarily during the second year. A similar result was found for non-healing wounds (not shown). By contrast, the prevalence of DVT/PE generally did not differ significantly among the three groups.

The longitudinal random intercept models indicate that the associations between injury group and wound complications varied by postinjury year when adjusting for covariates. Specifically, injury group and postinjury year interactions were significant for both diagnoses of infections and osteomyelitis after adjusting for covariates (*p* < 0.05; results not shown). For the early amputation group, there was an increased likelihood of any infection relative to limb salvage, but only during the first year postinjury. In contrast, the late amputation group had a significantly increased likelihood of any infection relative to limb salvage during the first three years postinjury. For osteomyelitis, early amputation was associated with a decreased likelihood relative to limb salvage, primarily during the third year postinjury, while the late amputation group had an increased likelihood for osteomyelitis only during the first 2 years relative to limb salvage. Regarding nonunion fractures, although there was no significant interaction, early amputation had significantly reduced ORs relative to late amputation and limb salvage during each of the first three years (*p*’s < 0.05). The fourth year results for osteomyelitis and nonunion fractures should be interpreted with caution because the small number of cases that year (less than 5% for each group) may have obscured the interaction effect.

### Other Physical Health Complications

#### Four-year prevalence

Referring back to Table 2, over 90% of patients in all groups had at least one pain-related diagnosis and at least one diagnosis for any of the musculoskeletal conditions during the first four years postinjury. The early amputation group (34%) had significantly lower prevalence for a diagnosis of late effects of musculoskeletal injury than did the late amputation (65%) and limb salvage groups (48%; *p*’s < 0.05). Early amputation (10%) also had a significantly lower prevalence for osteoarthritis than the late amputation or limb salvage groups (both 21%; *p*’s < 0.05; the anatomical location for 85% of all osteoarthritis diagnoses was a lower limb). By contrast, the early amputation group (16%) had a higher prevalence for osteoporosis than the late amputation group (8%) and significantly higher than the limb salvage group (2%; *p* < 0.05). For more specific diagnoses, nearly half of the early and late amputation groups had lumbago, with the limb salvage group having a significantly lower prevalence than the late amputation group (*p* < 0.05). Finally, there were no significant differences in the prevalence of diagnosis of hypertension among the unilateral groups.

Table 3 includes the results of the regression models for the occurrence of physical health complications during the first four years. After adjusting for covariates, the early amputation group generally had significantly reduced ORs for diagnoses of any musculoskeletal disorder, pain, and osteoarthritis relative to the limb salvage group. Though not shown in Table 3, the likelihood for these same complications in the early amputation group was also significantly lower than in the late amputation group. By contrast, early amputation had a significantly increased likelihood of osteoporosis relative to limb salvage. Blast injury was associated with an increased likelihood of pain and osteoarthritis, ISS with increased musculoskeletal diagnoses, age with increased pain and osteoarthritis, and injury year with decreased musculoskeletal diagnoses and pain.

#### Longitudinal results

**Fig 2:**
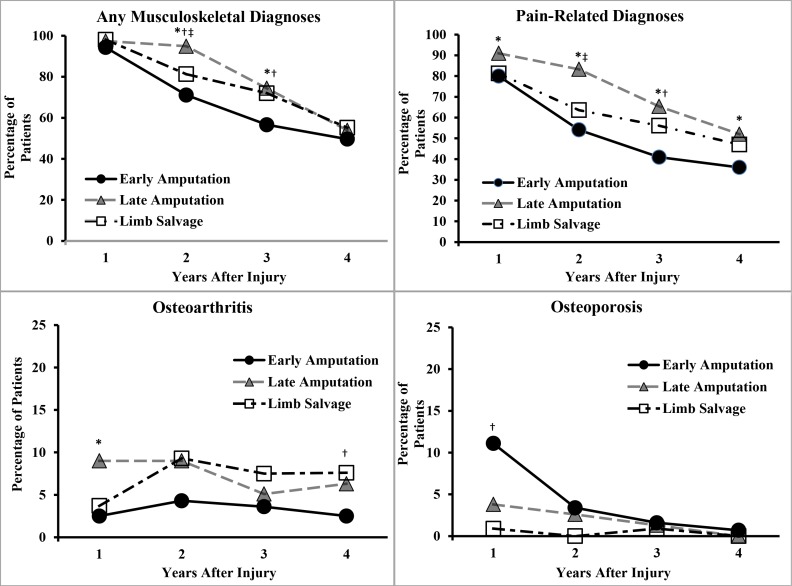
Prevalence of Selected Physical Health Complications by Time After Injury. Statistically significant difference between groups, using chi-square or Fisher’s exact test as appropriate, p < 0.05 were *early amputation versus late amputation, †early amputation versus limb salvage, and ‡late amputation versus limb salvage. (Table 3 shows where injury group was significantly associated with outcomes after adjusting for covariates).

Fig 2 shows that the prevalence for diagnoses of any musculoskeletal diagnosis, pain, and osteoporosis declined significantly across the first four years (*p* < 0.01). There was a small but statistically significant increase in the prevalence of osteoarthritis between years one and two (*p* < 0.05). However, there was no significant change in prevalence of osteoarthritis subsequently during years three and four. We observed a similar decline in the prevalence of late effects of injury (*p* < 0.01; data not shown in the figure). Interestingly, Fig 2 indicates a significantly higher prevalence for osteoporosis for early amputation than for limb salvage only during the first year. The prevalence of lumbago and hypertension did not change significantly over time (data not shown in the figure).

The longitudinal models also showed significant interactions between injury group and postinjury year only for diagnoses of musculoskeletal disorder and late effects of injury (*p* < 0.05; results not shown). First, for musculoskeletal diagnoses, early amputation had a significantly reduced likelihood relative to limb salvage during the second and third year postinjury (*p*’s < 0.05), while late amputation had a significantly increased likelihood during the second year (*p* < 0.05). Second, for late effects of injury diagnoses, early amputation showed a significantly reduced likelihood relative to limb salvage during the first three years post injury (*p*’s < 0.05), while late amputation showed an increased likelihood only during the first two years (*p*’s < 0.05).

### Psychological Disorders

#### Four-year prevalence

The percentage of patients with at least one diagnosis for a psychological disorder ranged from 82% to 89% and did not differ significantly between the three groups (Table 2). However, the early amputation group had significantly fewer diagnoses (mean/median = 2.7/3.0) than late amputation (mean/median = 3.3/4.0, *p* < 0.05). The limb salvage group also had fewer diagnoses (mean/median = 2.8/3.0) than late amputation. The prevalence of a PTSD diagnosis did not differ significantly among the three groups (49% to 58%; Table 2). Similarly, there were no group differences in the prevalence of diagnosed adjustment disorder (43% to 53%). For mood disorders, early amputation (39%) and limb salvage groups (36%) had significantly lower prevalence than did late amputation (55%; *p* < 0.05). For both anxiety and substance abuse diagnoses, early amputation had a significantly lower prevalence than late amputation (*p*’s < 0.05). Though not presented in Table 2, a similar association was found for tobacco use disorder. In summary, late amputation was associated with the highest prevalence of all groups for diagnoses of all six psychological disorders, and with significantly higher prevalence of mood, substance abuse, anxiety, and tobacco use disorder than the early amputation and/or limb salvage groups (*p*’s < 0.05).

Table 4 shows the results of the regression models of psychological diagnoses for the first four years postinjury. After adjusting for covariates, the late amputation group had a significantly increased likelihood of mood, adjustment, and other psychological disorders relative to limb salvage (*p*’s < 0.05). Late amputation was also associated with an increased likelihood of substance abuse relative to early amputation (results not shown).

**Table 4:** Summary Results for Overall (4 Year) Regression Models for Psychological Disorders.

Independent Variables	Postinjury Outcomes Variables OR (95% CI)					
	PTSD	Mood	Adjustment	Anxiety*	Substance Abuse	Other^†^
**Injury Group**						
** Limb salvage (ref)**	–	–	–	–	–	–
** Early amputation**	NS	NS	NS	NS	NS	NS
** Late amputation**	NS	2.83	2.36	NS	NS	2.66
		(1.31–6.09)	(1.20–4.62)			(1.49–4.77)
**Blast (vs. no blast, ref)**	NS	NS	NS	0.39	NS	2.29
				(0.20–0.76)		(1.37–3.86)
**ISS (log)**	NS	NS	NS	NS	NS	1.98
						(1.46–2.96)
**Age, yr**	0.93	NS	–	NS	0.94	NS
	(0.89–0.98)				(0.89–0.99)	
**Injury year (2001/5, ref vs. 2006/8)**	NS	NS	0.38	0.53	0.47	0.42
			(0.26–0.55)	(0.36–0.79)	(0.27–0.83)	(0.31–0.58)
**Preinjury psychological diagnosis**	NS	NS	NS	NS	NS	NS

Traumatic brain injury and injury location (above vs. below knee) were not significant in the models.

*Variables with significant injury group × time interaction (see results section for description of the interactions).

†Other psychological disorders included postconcussion syndrome, and cognitive and sleep disorders.

Substance abuse: late amputation had significantly increased OR relative to early amputation (OR = 2.55 [1.17–5.54]).

Anxiety: late amputation had significantly increased OR relative to early amputation (OR = 2.39 [1.34–4.25]).

PTSD: early amputation had significantly reduced OR relative to limb salvage (OR = 0.39 [0.15–0.97]) only during year 1, although the injury group × time interaction was not significant.

CI = confidence interval, ISS = Injury Severity Score, NS = not significant, OR = odds ratio, PTSD = posttraumatic stress disorder.

#### Longitudinal analysis

**Fig 3:**
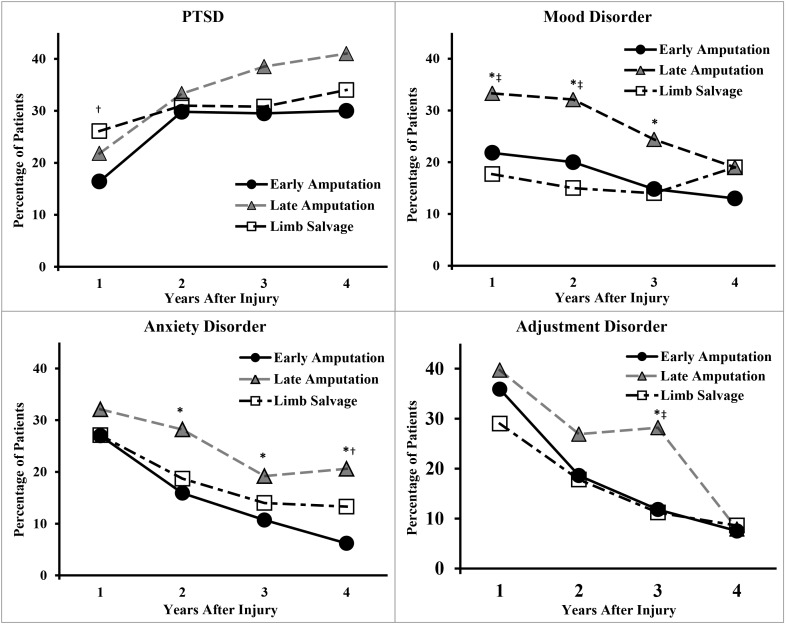
Prevalence of Selected Psychological Disorders by Time After Injury. Statistically significant difference between groups, using chi-square or Fisher’s exact test as appropriate, p < 0.05 were *early amputation versus late amputation, †early amputation versus limb salvage, and ‡late amputation versus limb salvage. (Table 4 shows where injury group was significantly associated with outcomes after adjusting for covariates). PTSD = posttraumatic stress disorder.

Fig 3 shows that the prevalence of PTSD diagnoses increased significantly between Year 1 and Years 2 through 4 (*p*’s < 0.01). By contrast, the prevalence of mood, anxiety, and adjustment disorder decreased significantly after Year 1 (*p’*s< 0.01). Diagnoses for tobacco use disorder (Year 1: 24% vs. Year 4: 12%) and other psychological disorders (e.g., postconcussion syndrome) also decreased significantly over time (data not shown in the figure; *p*’s < 0.01). There was no significant change in the prevalence of substance abuse diagnosis over the first four years.

The longitudinal regression models also showed significant interactions between injury group and postinjury year for diagnoses of anxiety (results not shown). Specifically, early amputation had significantly reduced likelihood of anxiety disorder relative to limb salvage or late amputation only during Years 2 and 4. Although PTSD was not significantly associated with injury group in the overall models, early amputation had a statistically significantly (60%) reduction in the likelihood of PTSD relative to limb salvage only during the first year after injury.

## Discussion

### Clinical Implications

This study is one of the first to integrate military and VA health data to follow the clinical outcomes of patients with severe combat extremity injuries. After adjusting for covariates, early amputation was associated with reduced likelihood for wound complications, osteomyelitis, musculoskeletal disorders, and some psychological disorders compared with late amputation or limb salvage. This was particularly evident during the first two years after injury. An important exception was that early amputation was associated with an increased likelihood of developing osteoporosis during the first year after injury. The study also provides some of the first results on secondary physical and mental health conditions affecting patients recovering from combat extremity injury [9]. For a relatively young study cohort, there was a generally high prevalence of pain, musculoskeletal disorders, osteoarthritis, substance abuse, tobacco abuse, and hypertension [28–29]. Notably, the prevalence of PTSD increased after the first year postinjury for all groups.

Previous reports have emphasized the need for health outcomes data to inform and improve treatment and rehabilitation of patients with serious leg injuries [4–9,30,31]. Our previous [5] and present study findings further support the need to investigate and refine existing clinical treatment pathways. For example, our results indicate the need for prolonged wound surveillance over years to manage infectious complications including osteomyelitis (Fig 1). This was particularly evident for the late amputation and limb salvage groups. For patients in all study groups, 10% to 20% had some diagnosis of infection even several years after injury. The results also showed that regular screening and prophylaxis for DVT and PE are important during the first year after injury for all groups.

The high prevalence of various physical health complications, particularly musculoskeletal and pain diagnoses, supports the need for prolonged follow-up, especially for the late amputation and limb salvage groups (Fig 2). More research is needed to describe the anatomic location and specific types of adverse musculoskeletal diagnoses (e.g., overuse injuries) observed in the present study. Nearly half of the present study cohort had diagnoses of lumbago, but these diagnoses were not independently associated with any injury group. The diagnoses may have been a direct result of injury or a secondary effect of altered gait following serious leg injury. Further research efforts should follow back pain beginning soon after injury because it may exacerbate existing functional limitations related to serious leg injuries.

There is also the need to describe the use of pain medications over time given the high rates of pain diagnoses for all groups. A related challenge is to identify the specific types and intensity of activities, such as strength conditioning, ambulation using prosthetics, swimming, and higher-level sports that might help or hinder the development of pain and/or musculoskeletal disorders following combat amputation and limb salvage. The high prevalence of musculoskeletal and pain diagnoses emphasizes the need for long-term programs to engage patients in training to minimize alterations in gait that typically follow serious lower extremity injuries [9,32]. Providers and patients should also carefully maintain or adjust their prostheses or orthoses for optimal function [9] and manage lifestyle and activities as appropriate to minimize secondary complications including pain, musculoskeletal disorders, obesity, and tobacco use [33].

The present results and one previous study [34] indicate the need for interventions to treat osteoporosis during the first year following early amputation. This is likely related to the fact that patients have limited ability to bear weight on their lower limbs for weeks or months prior to prosthetic training. Osteoporosis, along with the increased likelihood of falling during prosthetic training, may increase risk for limb fractures. Providers may consider implementing supplements and/or prescription medications as appropriate to sustain bone density until patients resume walking or obtain lower limb prostheses. The substantial decline in the prevalence of osteoporosis after the first year suggests that this condition improves after initial prosthetic fitting, weight-bearing activities, and daily ambulation (Fig 2).

The prevalence for some other physical health complications appears to be relatively high considering the present cohort averaged approximately 30 years of age at four years postinjury [35,36]. Previous reviews indicated elevated rates of musculoskeletal injuries following traumatic amputation [9]. The CDC estimates osteoarthritis in 13.9% of all adults 25 to 65 years old [28]. By comparison, approximately 20% of the late amputation and limb salvage groups in the present study had an osteoarthritis diagnosis by age 25 to 35 years. A study of deployed service members found that the prevalence of osteoarthritis increased significantly with age, particularly after age 35, relative to age-matched civilians [36]. Consequently, the present study cohort, who generally was not yet 35 years old, may be at a high risk for the development of osteoarthritis over the next five to 10 years. A survey study found that the three year incidence for hypertension was 6.1% among combatants with multiple deployments [29]. The present results indicate a 9%– 15% prevalence of hypertension for the patient groups over the first four years, suggesting that providers should monitor tobacco use and other cardiovascular risk factors.

A final and important clinical implication is the increase in prevalence of PTSD over time, particularly during the second year after injury (Fig 3). This finding indicates the need for regular and proactive screening for psychological disorders during routine health assessments by military and VA providers (e.g., primary care) for several years. This increase in PTSD may reflect the stress of discharge from military service and the transition to VA healthcare and/or quality-of-life issues [37–38]. We recently reported this finding for a smaller sample of 258 US servicemembers with combat related amputations [39]. The present results show that this finding extends to the larger population of patients with combat related amputations or limb salvage.

The present results and one recent survey study [5] help quantify the effectiveness of the well-developed military care programs for servicemembers with major limb amputations. These programs implement early and aggressive multi-specialty care and rapid return to activity, including advanced prostheses, particularly following early amputation. These factors may help to reduce the prevalence of some adverse physical and psychological diagnoses during the first several years postinjury [Figs 2 and 3]. By contrast, the clinical pathway for limb salvage was not well defined during the time of the present study (2001–2011).

### Study Strengths

The strengths of this study include the near-complete population of patients with combat-related amputations from 2001 through 2008 [18–20] and detailed casualty records from the EMED [11]. The casualty records provided ISS for all patients and allowed identification of a sample of patients with limb salvage injuries selected randomly from patients with serious extremity injuries in the EMED. Clinical records used for analysis of health outcomes were available for over 90% of patients each year through four years postinjury. The present collaboration between DoD and VA investigators and appropriate institutional review board agreements allowed this investigative team to integrate DoD and VA health data [15–16]. This collaboration permitted longitudinal retrospective analysis of numerous physical and psychological diagnoses at regular intervals for a relatively large study cohort.

### Study Weaknesses

The primary limitation of the present research is that it was a retrospective observational study comparing nonrandomized groups. The results do not assess patient mobility, functional capacity, or quality of life. A comprehensive understanding of health outcomes should include a population-based survey to evaluate patient functional mobility and quality-of-life outcomes over time. NHRC is presently enrolling patients in the Wounded Warrior Recovery Project to evaluate patient physical, psychological, and overall functional quality of life [40].

There are also limitations on the generality of the present results for patients treated with limb salvage. This study group was identified from a random sample of patients with serious lower-extremity injuries from the EMED database. As discussed, there are no well-established methods to identify the population of patients who underwent limb salvage following injuries in Iraq and Afghanistan. Therefore, we identified a sample of patients with leg-threatening injuries by applying criteria used in previous studies [5,6,22–24]. The present study did not specifically identify patients who underwent so-called “functional limb salvage.” There are likely many such patients who sustained extremity trauma that were not leg-threatening, but caused functional limitations (e.g., required significant therapeutic interventions such as orthoses). The ISS measure includes lower-limb amputation and limb salvage injury, but does not include other injuries to the lower limb(s), which may have contributed to outcomes. We are presently completing AIS coding for all injuries to various body regions, including upper and lower limbs, to improve statistical modeling in future studies. This will also allow calculation of alternative injury scoring methods such as the New Injury Severity Score or NISS [41].

Most patients in the early amputation group sustained traumatic amputations and were not candidates for limb salvage. This fact could limit the generality of the present results. Also, patients with late amputation could be analyzed as “failed” limb salvage [22]. However, it is not clear whether the late amputation group had injuries that would have qualified as leg threatening, according to the present and previous criteria [5,6,21–24], and some may even represent reconstructive amputations to optimize a patient’s functional mobility [21,42].

The prevalence reported for some of the health outcomes are based on clinical diagnoses, which may underestimate their true occurrence. However, the prevalence of key variables in our present and previous studies is similar to other studies that focused on a specific variable using detailed chart reviews (e.g., infections, osteomyelitis, and osteoporosis) [43–44]. Our finding of a lower prevalence of PTSD for amputation versus limb salvage is consistent with Doukis et al. [6]. The present results also show a consistent pattern across variables, such as higher rates for both adverse physical and psychological outcomes for late amputation and/or limb salvage versus early amputation. Some disorders may have overlapping symptoms such as PTSD and TBI, and this may have led to misdiagnosis [45]. The clinical diagnoses captured by this research did not include information on the severity of specific conditions (e.g., TBI, lumbago) or the anatomic location of other diagnoses (e.g., osteoarthritis).

The present research is a broad-based surveillance study including multiple physical and psychological variables at multiple points in time. Electronic records are the best available method to maintain consistent follow-up for an entire cohort over long periods. As discussed, our results provide initial data to inform and focus follow-up studies that might use chart reviews and/or prospective research designs.

### Next Steps

This study describes a relatively young cohort and the present results for the lower limb groups indicate a relatively high prevalence of other physical health complications. Future research will continue to track this cohort. There is also a need to identify their healthcare use, needs, and associated costs, including the transition of care between military and VA healthcare facilities. Similar surveillance studies should focus on the most recent patients who sustained amputations or serious leg injuries due to their service in Afghanistan from 2009 to 2014.

## Conclusions

The present study shows differences in the adverse physical and psychological health outcomes for combat-injured patients treated with early amputation, late amputation, and limb salvage over the first four years postinjury. This study also provides initial results to inform and optimize clinical treatment and rehabilitation pathways specific to the needs of these different patient groups. Early amputation was generally associated with similar or reduced physical and psychological disorders relative to successful limb salvage or late amputation. A notable exception was an increased likelihood of osteoporosis following early amputation. Most evident was that late amputation (>90 days postinjury) was significantly associated with relatively high prevalence of physical and psychological disorders. Over time, all groups showed relatively high rates of complications including musculoskeletal disorders, pain, tobacco use disorder, mood disorders, and PTSD. The present results indicate that some musculoskeletal and psychological disorders persist for at least four years postinjury and may increase in the long-term as these patients age. Patients with severe combat extremity injuries typically require prolonged rehabilitation and, in the case of individuals with amputation and many limb salvage, lifelong prosthetic or orthotic care [31,46]. Future research should continue to track their military and VA health data outcomes over the long-term.
