# Correction: A Comparison of Four-Year Health Outcomes following Combat Amputation and Limb Salvage

**DOI:** 10.1371/journal.pone.0173214

**Published:** 2017-02-27

**Authors:** Ted Melcer, Jay Walker, Vibha Bhatnagar, Erin Richard, V. Franklin Sechriest II, Michael Galarneau

There is an error in the last sentence of the Results section. The correct sentence is: Although PTSD was not significantly associated with injury group in the overall models, early amputation had a statistically significant (Odds ratio = 0.39 [95% confidence interval: 0.15–0.97]) reduction in the likelihood of PTSD relative to limb salvage only during the first year after injury.

## References

[1] Melcer T, Walker J, Bhatnagar V, Richard E, Sechriest VF II, Galarneau M (2017) A Comparison of Four-Year Health Outcomes following Combat Amputation and Limb Salvage. PLoS ONE 12(1): e0170569 doi: 10.1371/journal.pone.0170569 28122002 10.1371/journal.pone.0170569 PMC5266314

